# A Novel Multimodal Therapy for Anaplastic Thyroid Carcinoma: ^*125*^I Seed Implantation Plus Apatinib After Surgery

**DOI:** 10.3389/fendo.2020.00207

**Published:** 2020-04-22

**Authors:** Yiqi Niu, Zheng Ding, Xianzhao Deng, Bomin Guo, Jie Kang, Bo Wu, Youben Fan

**Affiliations:** ^1^Department of Thyroid-breast-hernia Surgery, Shanghai Jiao Tong University Affiliated Sixth People's Hospital, Shanghai, China; ^2^The International Peace Maternity and Child Health Hospital, School of Medicine, Shanghai Jiao Tong University, Shanghai, China

**Keywords:** anaplastic thyroid cancer, apatinib, ^125^I seed implantation, brachytherapy, multimodal therapy

## Abstract

Anaplastic thyroid cancer is known to be the most lethal malignancy among endocrine tumors for its extremely limited survival rate after diagnosis. As a result of this poor survival prognosis, multimodal therapy is currently under investigation to address this global concern. In this reported case, the ^125^I seed implantation and vascular endothelial growth factor receptor-2 (VEGFR-2) inhibitor apatinib were co-applied to treat a 49-year-old woman with anaplastic thyroid cancer. After the patient began apatinib administration and underwent ^125^I seed implantation twice, the tumor size shrank successfully. After a follow-up of 13 months since the initial diagnosis of anaplastic thyroid cancer, the patient survived with a stable disease pathology. In conclusion, this study supports ^125^I seed implantation and apatinib as effective therapeutic alternatives for inoperable anaplastic thyroid cancer patients.

## Introduction

Based on clinical definition, anaplastic thyroid cancer (ATC) is an undifferentiated thyroid cancer demonstrating epithelial differentiation with immunohistochemical or ultrastructural detectable features (1). In all thyroid cancers, most differentiated thyroid cancers (DTC) have an optimistic prognosis after standard treatment, while ATCs exhibit a median survival of only 3–10 months, with only 20% (2, 3) of patients reaching the full year, which accounts for more than 50% of deaths of all thyroid cancers (4). The small numbers of diagnosed cases, poor prognosis, and insufficient follow-up time cause difficulty in generating a standardized treatment protocol. Although multidisciplinary therapies have been adopted, the median survival time did not remarkably change over half a century (5).

Considering its malignancy, all ATC cases are classified as stage IV by the American Joint Committee on Cancer (AJCC) (6). IVa represents all the tumors which are limited in the gland, IVb stands for the tumors with extrathyroidal extension, and IVc describes detected distant metastasis. Thus, the National Comprehensive Cancer Network (NCCN) Guidelines (7) recommend a total thyroidectomy plus selective lymph nodes dissection of the invaded region in ATC which is viable for resection. Postoperative radiotherapy and/or chemotherapy are optional. However, most ATC patients suffer from inoperable tumors, and with our case, multimodal therapy would provide a benefit to improving the patient's life quality and preventing further metastases. Given the poor response to conventional therapies, ATC patients are appropriate candidates for neoadjuvant therapies, including ^125^I seed implantation and molecule-targeted drugs, proven to be effective in other tumors (1, 8–10). Apatinib is a small molecule, broad antineoplastic targeted drug, which acts as a selective vascular endothelial growth factor receptor-2 (VEGFR-2) inhibitor.

Herein, we report a case of an ATC patient who received a novel multimodal treatment after two surgeries, with a favorable survival of 13 months.

## Case Report

**Figure 1:**
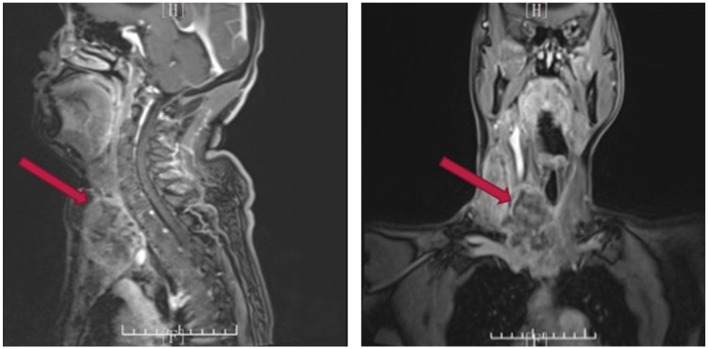
Cervical enhanced MRI indicating tumor recurrence.

A 49-year-old woman who had a mass in her right thyroid for 3 months was referred to our department. The patient had recently undergone a right lateral thyroidectomy in another hospital. After histopathological testing, papillary thyroid carcinoma was confirmed. However, the mass in the right anterior neck increased rapidly in size within 1 month again. The mass was hard and unmovable. Cervical enhanced Magnetic Resonance Imaging (MRI) displayed a solid tumor with internal heterogeneity, 5.4 × 6.0 cm in size, compressing the trachea leftwards, as well as multiple right cervical lymph nodes enlargements around the carotid sheath (Figure 1). Positron Emission Tomography-Computed Tomography (PET-CT) revealed a significant 18F-fluorodeoxyglucose (18F-FDG) uptake in the mass and right cervical lymph nodes. No distant metastasis was observed. The fine needle aspiration cytology (FNAC) result was suggestive of poorly differentiated thyroid carcinoma with an undetermined histological type.

**Figure 2:**
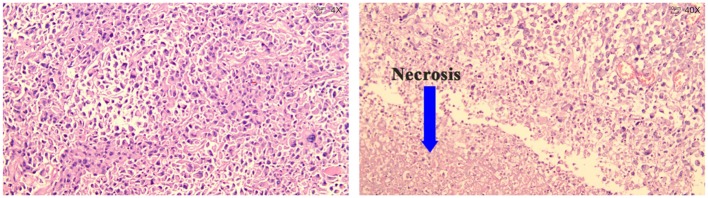
Hematoxylin Eosin staining of ATC with massive necrosis.

Based on the clinical features above, we identified thyroid cancer reappearing. Moreover, the tumor type was also shown to be deteriorated. The tumor adhered tightly to the trachea and left common carotid and invaded the right sternocleidomastoid muscle. Thus, palliative surgery was performed, resecting the majority of the tumor, based on the aforementioned guidelines (7). The histopathological findings of the surgical specimens showed ATC with massive necrosis (Figure 2), and the final stage was confirmed as IVb (6). The sample from the surgery was tested for molecular alterations and the results were BRAF mutation-negative, TERT promoter mutation-positive (C228T), and RET fusion (NCOA4_1:RET_12), which were proved relevant to aggressive tumor characteristics, tumor recurrence, and patient mortality (11, 12). We then initiated an oral administration of apatinib (500 mg/day) after obtaining informed consent. The most common side effect of apatinib is high blood pressure; thus, the patient was advised to continue taking antihypertensive drugs because of her history of hypertension, and was advised to monitor blood pressure at home.

**Figure 3:**
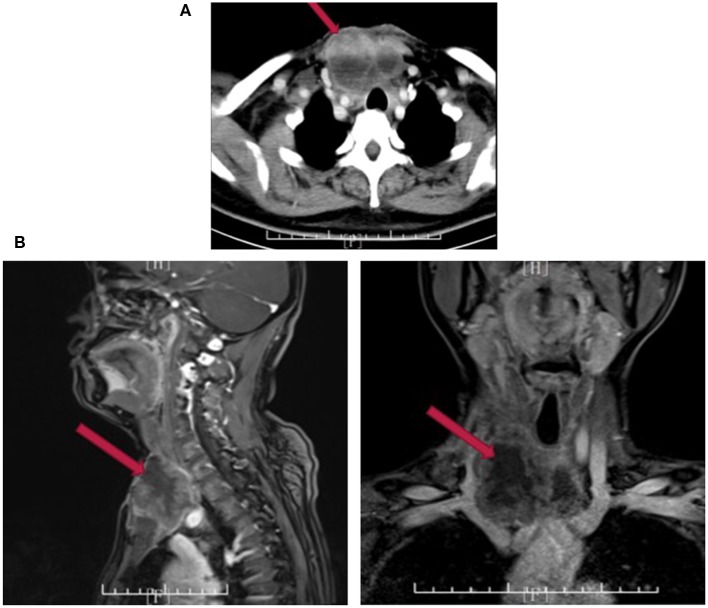
Cervical enhanced CT **(A)** and MRI **(B)** 20 days after palliative surgery.

Twenty days after administration of apatinib, the patient came back to our outpatient department. Both cervical enhanced computer tomography (CT) and MRI showed a mass of 6.3 × 5.4 cm in the right anterior neck, extending to superior mediastinum (Figure 3). As a matter of fact, the patient could barely benefit from surgery again. Subsequently, we decided to turn our attention to radiation therapy, which includes traditional external beam radiation therapy (EBRT) and internal beam radiation therapy. ^125^I seed implantation is the most used method of brachytherapy. For less side effect and cost, ^125^I seed implantation (a number of 40 particles) was performed under the guidance of the interventional radiologist.

**Figure 4:**
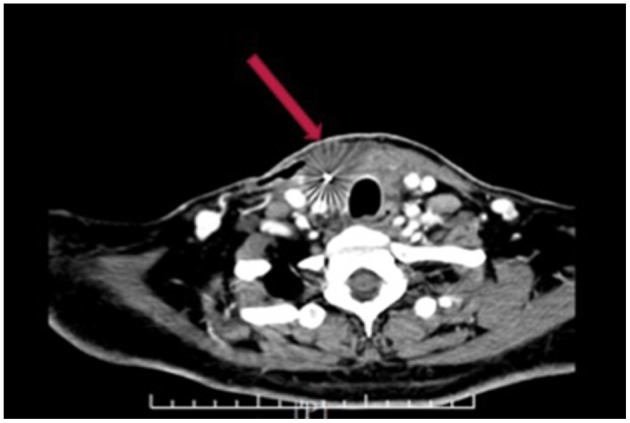
Cervical enhanced CT 1 month after first ^125^I seed implantation.

One month later, cervical enhanced CT (Figure 4) showed a reduced tumor (3 × 5.4 cm), which meant ^125^I seed implantation appeared to be effective, thus the ^125^I seed implantation (a number of 40 particles) was additionally performed again.

After 6 months, the disease was stable, and there was no severe adverse reaction against apatinib or ^125^I seed except for a non-healing surgical incision. In order to improve the incision's appearance and life quality, dermepenthesis was advised for the patient. Pedicled pectoralis major muscle without flap was grafted to the defective neck and skin was collected from the thigh, which were designed to preserve the appearance of the breast. Two weeks after surgery, the pedicled muscle survived and the incision was gradually healing. Presently, it has been 13 months since the novel multimodal therapies, and the tumor size has no obvious enlargement.

## Discussion

Given the international concern of thyroid cancer and its increased morbidity for all pathological types including ATC (5), better treatment standardization must be established for patient survival and quality of life. Sugitani et al. (13) developed a prognostic index (PI) for ATC on the basis of four adverse prognostic factors: (1). Acute symptoms (duration of severe complaints such as dysphonia, dysphagia, dyspnea, and rapid tumor growth <1 month); (2). Leukocytosis (leukocyte counts ≥ 10,000); (3). Tumor size larger than 5 cm; (4). Distant metastasis. In their study, patients would achieve a PI score from 1 to 4, and patients with PI = 1 experienced a 62% survival rate for 6 months, whereas no patients with PI = 3 survived longer than 6 months, and all patients with PI = 4 died within 3 months. The PI score of our case was 2; fortunately, the patient survived for 13 months because of the ^125^I seed implantation plus the apatinib therapies after surgery.

It has been reported that about 50% of ATC patients have distant metastases at presentation (14); however, the more common causes of deaths are associated with complications resulting from local invasion, such as acute massive hemorrhage, air obstruction, or circulatory failure on account of compression in mediastinal vasculature (15). As ATC progresses, local invasion would bring about various compressive symptoms including dyspnea, dysphagia, baryodynia, and trachyphonia that often deprive the chance of surgery.

In view of rapid recurrence, radical resection even including laryngectomy or esophagectomy has little to no benefit to survival outcomes in ATC IVb and IVc (16, 17) patients. Therefore, multimodal treatment for ATC patients with PI scores no more than 1 was advised while patients with PI score no <3 were advised to avoid aggressive treatment (13, 18). Multimodal therapy provides new reasonable approaches to achieve symptom improvement and regional control for ineradicable surgery. Prasongsook et al. (19) reported a cohort study of ATC patients between multimodal therapy (radiation therapy plus chemotherapy) and palliative treatment, and the median overall survival was 21 vs. 3.9 months among non-metastatic ATC patients. However, about 60% of patients receiving multimodal therapy required hospitalization because of attendant therapy-related toxicities and at least temporary feeding tube placement. Hence, tradeoff between attaining prolonged overall survival and imposed collateral toxicities should be evaluated. Although multimodal therapy appears to convey longer survival in ATC patients, effective and tolerable approaches are needed in terms of minimizing side effects and maintaining quality of life.

There has been controversy regarding chemotherapy for ATC with no sufficiently supportive evidence. It has been reported that chemotherapy combined with EBRT after radical surgery showed prolonged survival in IVc patients, and combined paclitaxel and pemetrexed was the only regimen associated with improved overall survival (14), whereas other chemotherapeutic drugs have not brought significantly prolonged survival (20). These treatments have serious side effects, such as myelo-suppression, trichomadesis and gastrointestinal reactions, and less sensibility when compared with targeted drugs. However, targeted drugs for ATC are mostly at the experimental stage. Taking DTC for reference, common targeted drugs can be divided into BRAF-directed inhibitors, multikinase inhibitors, and mTOR inhibitors (21). Ohkubo et al. (22) described the case of an ATC patient with lung metastasis, whose pulmonary nodules became hollow after lenvatinib (a multikinase inhibitor) administration. Subbiah et al. (23) used dabrafenib and trametinib (BRAF-directed inhibitors) for locally advanced or metastatic BRAF V600E–mutant ATC patients, receiving an overall response rate of 69% and one-year survival of 80%, which can also be a reminder of trying combined targeted drugs and routine genetic mutations tests in ATC patients for better medical guidance. As for our case, results of genetic molecular tests suggested a RET fusion, an oncogenic driver found activated in several cancers including DTC, medullary thyroid cancer, and non-small cell lung cancer (24). Multiple selective RET inhibitors are under clinical trials (25). For instance, BLU-667 is a potent RET inhibitor designed to target RET directly, which has successfully induced tumor regression without notable toxicity in patients harboring RET alterations, exhibiting enormous therapeutic potential (26). Finally, we chose apatinib for its broad antineoplastic efficacy (27–30) and fewer side effects compared with sorafenib (30). Apatinib, a multikinase inhibitor, specifically targets VEGFR-2 to partially block the pathways of mitogenic and angiogenic, by which it plays an essential role in tumor oncogenesis and metastasis (31).

Although ATC is highly resistant to radiation (32), it was reported that postoperative radiotherapy could be beneficial for regional control (33). On the other hand, patients must bear with large dose, high frequency, long duration and evident side effects in traditional EBRT, which may be overcome by ^125^I seed implantation to some extent. Radioactive particles that are directly implanted into the tumor induce radiation damage continuously, while sharply decreasing radioactive dosage causes adjacent tissue to sustain little harm. ^125^I seed implantation therapy has been a standard schedule for early prostate cancer recommended by NCCN guidelines (34).

In conclusion, we reported a case of an ATC patient who received two surgeries, two ^125^I seed implantation and apatinib administration. The ^125^I seed implantation and molecular-targeted drug apatinib have resulted in local relief in tumor size and exhibited a powerful anti-neoplastic effect, which can be proven as feasible therapeutic alternatives for inoperable ATC patients.

## Data Availability Statement

All datasets generated for this study are included in the article/supplementary material.

## Ethics Statement

Written informed consent was obtained from the individual(s) for the publication of any potentially identifiable images or data included in this article.

## Author Contributions

BW and YF conceived the idea of this essay and were responsible for the treatment. YN, XD, JK, and BG participated in the treatment and collected the case history. YN wrote the case report. ZD revised the manuscript.

## Conflict of Interest

The authors declare that the research was conducted in the absence of any commercial or financial relationships that could be construed as a potential conflict of interest.
